# Supplementary material for: Role of the Sodium-Dependent Organic Anion Transporter (SOAT/SLC10A6) in Physiology and Pathophysiology
Source: Int J Mol Sci. 2023 Jun 8;24(12):9926. doi: 10.3390/ijms24129926 (PMC10298714; doi:10.3390/ijms24129926)
Supplement: Supplementary file 1 [file ijms-24-09926-s001.zip › ijms-2390183-supplementary.pdf]

Role of the sodium-dependent organic anion transporter (SOAT/SLC10A6) in physiology and pathophysiology

Marie Wannowius [1], Emre Karakus [1], Zekeriya Aktürk [2], Janina Breuer [1], and Joachim Geyer [1,*]

1 Institute of Pharmacology and Toxicology, Faculty of Veterinary Medicine, Biomedical Research Center Seltersberg (BFS), Justus Liebig University of Giessen, Schubertstr. 81, 35392 Giessen, Germany; marie.t.wannowius@vetmed.uni-giessen.de (M.W.); emre.karakus@vetmed.uni-giessen.de (E.K.); janina.breuer@vetmed.uni-giessen.de (J.B.); joachim.m.geyer@vetmed.uni-giessen.de (J.G.)

2 General Practice, Faculty of Medicine, University of Augsburg, 86159 Augsburg, Germany; zekeriya.akturk@gmail.com

* Correspondence: joachim.m.geyer@vetmed.uni-giessen.de; Tel.: +49-641-9938404

Supplementary Material

**Supplementary Table S1**	Compounds with low or no inhibitory effect on SOAT.
**Supplementary Table S2**	Number of analyzed tissues for RNA-seq data generated by GTEx (exported 2022/03).
**Supplementary Table S3**	Internet sources of SOAT (SLC10A6) IHC images accessed via the Human Protein Atlas (V21.0.proteinatlas.org).

Supplementary Table S1. Compounds with low or no inhibitory effect on SOAT.

Test compound	Inhibitor IC_{50}
**Bile acids**	
Dehydrocholic acid	n.d. [1]
**Naphtyl derivatives**	
α-Naphthylamine	n.d. [1]
α-Naphthylisothiocyanate	n.d. [1]
α-Naphthylphosphate	n.d. [1]
**Non-steroidal organosulfates**	
2-Propylsulfate	n.d. [1]
5-Sulfooxymethylfurfural	n.d. [1]
Ethylsulfate	n.d. [1]
Hydroquinone sulfate	n.d. [1]
Indoxylsulfate	n.d. [1]
Phenylethylsulfate	n.d. [1]
Phenylsulfate	n.d. [1]
**Steroids**	
Aldosterone	>1000 µM [48]
Androstendione	>1000 µM [48]
Androsterone	>1000 µM [48]
Cholesteryl sulfate	>1000 µM [48]
Corticosterone	>1000 µM [48]
Cortisol	>1000 µM [48]
Digitoxigenin	>1000 µM [48]
Digoxin	>1000 µM [48]
Dihydrotestosterone	>1000 µM [48]
17β-Estradiol	>1000 µM [48]
Estrone	>1000 µM [48]
Ethinylestradiol	~900 µM [48]
Finasteride	>1000 µM [48]
Ouabain	>1000 µM [48]
Prednisolone	>1000 µM [48]
Prednisone	>1000 µM [48]
Progesterone	>1000 µM [48]
Testosterone	>1000 µM [48]
**Others**	
Ezetimibe	n.d. [14]
S 0381	~1000 µM [48]
S 0925	~1000 µM [48]
S 8005	~1000 µM [48]
Wortmannin	>1000 µM [48]

n.d. = not determined. The following [^{3}H]DHEAS substrate concentrations were used: 2.5 µM [1], 0.2 µM [14, 48]. Where no IC_{50} values were determined, 25 µM [1] or 100 µM [14] maximal inhibitory concentrations were used.

Supplementary Table S2. Number of analyzed tissues for RNA-seq data generated by GTEx (exported 2022/03).

Tissue	Tissue expression data obtained via GTEx	Number of samples
**Adipose tissue**	Adipose - Subcutaneous	663
	Adipose - Visceral (Omentum)	541
**Adrenal gland**	Adrenal Gland	258
**Amygdala**	Brain - Amygdala	152
**Breast**	Breast - Mammary Tissue	459
**Caudate**	Brain - Caudate (basal ganglia)	246
**Cerebellum**	Brain - Cerebellar Hemisphere	215
	Brain - Cerebellum	241
**Cerebral cortex**	Brain - Anterior cingulate cortex (BA24)	176
	Brain - Cortex	255
	Brain - Frontal Cortex (BA9)	209
**Cervix**	Cervix - Ectocervix	9
	Cervix - Endocervix	10
**Colon**	Colon - Sigmoid	373
	Colon - Transverse	406
**Endometrium**	Uterus - Endometrium	16
**Esophagus**	Esophagus - Mucosa	555
**Fallopian tube**	Fallopian Tube	9
**Heart muscle**	Heart - Atrial Appendage	429
	Heart - Left Ventricle	432
**Hippocampus**	Brain - Hippocampus	197
**Hypothalamus**	Brain - Hypothalamus	202
**Kidney**	Kidney - Cortex	85
	Kidney - Medulla	4
**Liver**	Liver	226
**Lung**	Lung	578
**Nucleus accumbens**	Brain - Nucleus accumbens (basal ganglia)	246
**Ovary**	Ovary	180
**Pancreas**	Pancreas	328
**Pituitary gland**	Pituitary	283
**Prostate**	Prostate	245
**Putamen**	Brain - Putamen (basal ganglia)	205
**Retina**	Retina	105
**Salivary gland**	Minor Salivary Gland	162
**Skeletal muscle**	Muscle - Skeletal	803
**Skin**	Skin - Not Sun Exposed (Suprapubic)	604
	Skin - Sun Exposed (Lower leg)	701
**Small intestine**	Small Intestine - Terminal Ileum	187
**Spinal cord**	Brain - Spinal cord (cervical c-1)	159
**Spleen**	Spleen	241
**Stomach**	Stomach	359
**Substantia nigra**	Brain - Substantia nigra	139
**Testis**	Testis	361
**Thyroid gland**	Thyroid	653
**Urinary bladder**	Bladder	21
**Vagina**	Vagina	156

Supplementary Table S3. Internet sources of SOAT (SLC10A6) IHC images accessed via the Human Protein Atlas (V21.0.proteinatlas.org).

Normal Tissue	URL: www.proteinatlase.org...	Patient ID
**Breast**	/ENSG00000145283-SLC10A6/tissue/breast#img	3544
**Bronchus**	/ENSG00000145283-SLC10A6/tissue/bronchus#img	3482
**Cervix**	/ENSG00000145283-SLC10A6/tissue/cervix#img	3313
**Esophagus**	/ENSG00000145283-SLC10A6/tissue/esophagus#img	3197
**Nasopharynx**	/ENSG00000145283-SLC10A6/tissue/nasopharynx#img	2688
**Oral mucosa**	/ENSG00000145283-SLC10A6/tissue/oral+mucosa#img	2550
**Prostate**	/ENSG00000145283-SLC10A6/tissue/prostate#img	2053
**Skin**	/ENSG00000145283-SLC10A6/tissue/skin#img	3403
**Stomach**	/ENSG00000145283-SLC10A6/tissue/stomach#img	2130
**Tonsil**	/ENSG00000145283-SLC10A6/tissue/tonsil#img	2615
**Vagina**	/ENSG00000145283-SLC10A6/tissue/vagina#img	2480
**Cancer Tissue**	**URL: www.proteinatlase.org...**	**Patient ID**
**Pancreas Carcinoid**	/ENSG00000145283-SLC10A6/pathology/carcinoid#img	2618
**Lung Cancer**	/ENSG00000145283-SLC10A6/pathology/lung+cancer#img	2354
**Skin Cancer**	/ENSG00000145283-SLC10A6/pathology/skin+cancer#img	3520
**Cervix Cancer**	/ENSG00000145283-SLC10A6/pathology/cervical+cancer#img	470
**Head and Neck Cancer**	/ENSG00000145283-SLC10A6/pathology/head+and+neck+cancer#img	1743
**Urothelial Cancer**	/ENSG00000145283-SLC10A6/pathology/urothelial+cancer#img	1871

References

1. Geyer, J.; Döring, B.; Meerkamp, K.; Ugele, B.; Bakhiya, N.; Fernandes, C.F.; Godoy, J.R.; Glatt, H.; Petzinger, E. Cloning and functional characterization of human sodium-dependent organic anion transporter (SLC10A6). *J Biol Chem* **2007**, *282*, 19728-19741, doi:10.1074/jbc.M702663200.

14. Grosser, G.; Müller, S.F.; Kirstgen, M.; Döring, B.; Geyer, J. Substrate Specificities and Inhibition Pattern of the Solute Carrier Family 10 Members NTCP, ASBT and SOAT. *Front Mol Biosci* **2021**, *8*, 689757, doi:10.3389/fmolb.2021.689757.

48. Grosser, G.; Baringhaus, K.H.; Döring, B.; Kramer, W.; Petzinger, E.; Geyer, J. Identification of novel inhibitors of the steroid sulfate carrier 'sodium-dependent organic anion transporter' SOAT (SLC10A6) by pharmacophore modelling. *Mol Cell Endocrinol* **2016**, *428*, 133-141, doi:10.1016/j.mce.2016.03.028.
